# RyR1-targeted drug discovery pipeline integrating FRET-based high-throughput screening and human myofiber dynamic Ca^2+^ assays

**DOI:** 10.1038/s41598-020-58461-1

**Published:** 2020-02-04

**Authors:** Robyn T. Rebbeck, Daniel P. Singh, Kevyn A. Janicek, Donald M. Bers, David D. Thomas, Bradley S. Launikonis, Razvan L. Cornea

**Affiliations:** 1 Department of Biochemistry, Molecular Biology, and Biophysics, University of Minnesota, Minneapolis, MN 55455 USA; 2 School of Biomedical Sciences, The University of Queensland, Brisbane, QLD 4072 Australia; 3 Department of Pharmacology, University of California at Davis, Davis, CA 95616 USA

**Keywords:** Biological fluorescence, High-throughput screening

## Abstract

Elevated cytoplasmic [Ca^2+^] is characteristic in severe skeletal and cardiac myopathies, diabetes, and neurodegeneration, and partly results from increased Ca^2+^ leak from sarcoplasmic reticulum stores via dysregulated ryanodine receptor (RyR) channels. Consequently, RyR is recognized as a high-value target for drug discovery to treat such pathologies. Using a FRET-based high-throughput screening assay that we previously reported, we identified small-molecule compounds that modulate the skeletal muscle channel isoform (RyR1) interaction with calmodulin and FK506 binding protein 12.6. Two such compounds, chloroxine and myricetin, increase FRET and inhibit [^3^H]ryanodine binding to RyR1 at nanomolar Ca^2+^. Both compounds also decrease RyR1 Ca^2+^ leak in human skinned skeletal muscle fibers. Furthermore, we identified compound concentrations that reduced leak by > 50% but only slightly affected Ca^2+^ release in excitation-contraction coupling, which is essential for normal muscle contraction. This report demonstrates a pipeline that effectively filters small-molecule RyR1 modulators towards clinical relevance.

## Introduction

In striated muscle, contraction requires an intracellular Ca^2+^-release event mediated by ryanodine receptors (RyR) that are embedded in the sarcoplasmic reticulum (SR) membrane. Dysregulation of skeletal (RyR1) and cardiac (RyR2) isoforms, via mutations or excess posttranslational modification, has been linked to severe muscle pathologies, including malignant hyperthermia (MH), central core disease, muscular dystrophy (MD), sarcopenia, catecholaminergic polymorphic ventricular tachycardia, heart failure, and more recently RyR2 has been recognized as a potentially significant contributor to diabetes and Alzheimer’s disease^1–9^. In most of these clinical indications, pathogenesis can be fueled by excess SR Ca^2+^ “leak” via RyR under resting cellular conditions, which leads to toxic intracellular basal [Ca^2+^] and insufficient SR Ca^2+^ load. As a result, RyR is intensely studied as a therapeutic target. Indeed, the therapeutic potential of pharmaceutically targeting RyR1-mediated SR Ca^2+^ leak in skeletal muscle has been shown in animal models of Duchenne MD, limb-girdle MD, and sarcopenia^5,10,11^. The therapeutic potential of targeting RyR2-mediated SR Ca^2+^ leak for treating heart failure and arrhythmia is also very well documented^12–16^. Additionally, targeting RyR2 (which is abundant in the brain^17,18^) may have therapeutic potential for treating neurodegenerative diseases.

To introduce a systematic and efficient approach for identifying novel small-molecule chemical scaffolds with potential to mitigate RyR1 dysfunction, we developed and implemented a high-throughput screening (HTS) assay that uses fluorescence lifetime (FLT) detection of FRET^19^. This assay was designed to identify compounds that bind to the RyR1 channel complex to allosterically correct its pathologically leaky state (without affecting normal channel function)^19^. This FRET-based method is based on monitoring RyR binding of fluorescently labeled variants of two known RyR modulators–the FK506 binding protein (FKBP) 12.6 and calmodulin (CaM). It has been shown that binding of CaM and FKBP to RyR can be allosterically influenced by small-molecule RyR modulators (e.g., dantrolene, K201, and S107) and RyR post-translational modifications^9,20–23^. Despite some controversy regarding the connection between RyR phosphorylation and FKBP association^24,25^, a growing consensus is that CaM and/or FKBP binding to RyR may provide direct insight into the RyR structural state that has been associated with Ca^2+^ leak^22,23,26–29^. We previously used this HTS platform in a 384-well format to screen a small collection of compounds approved for clinical use^19^. In that report, we identified a correlation between the effects of Hit compounds on the FLT readout and RyR1 activity at resting Ca^2+^. Four Hits that reduced FRET also increased RyR1 activity, measured using [^3^H]ryanodine binding to skeletal heavy SR membranes (HSR)^19^. Correspondingly, the one Hit (chloroxine) that increased FRET also displayed the desired functional effect, which is RyR1 inhibition at resting [Ca^2+^]^19^. In a phenotypic screen by another group, using HEK293 cells stably expressing a genetically encoded ER Ca^2+^ sensor and an MH-linked RyR1 mutant, three compounds in common with Hits from our structural screen were identified as potential RyR1 modulators^30^. However, in that phenotypic screen, chloroxine’s effect on the readout suggested increased RyR1 activity, and thus was inconsistent with our findings^19^.

Here, our goals were to implement and validate our HTS assay in 1536-well plates (the industry standard), expand the array of potential RyR1 leak inhibitors, and directly correlate our FRET assay (structural) with Hit effects in an RyR1 leak assay (functional) that uses mechanically skinned human muscle fibers. This recently reported breakthrough technology preserves the excitation-contraction (EC) coupling apparatus^31^, which is essential for compound validation, given that current HTS methods lack the dihydropyridine receptor (DHPR), which is a crucial *in vivo* RyR modulator in the context of EC-coupling. Overall, we demonstrate a screening pipeline for RyR1-targeted drug discovery and development.

## Results

### HTS performance

To confer compatibility with current HTS standards in industrial drug discovery facilities, we miniaturized our screening format from the previously established 384-well plates, requiring 50 μL sample per well^19^, to 1536-well plates, requiring 5 μL sample per well. The complete 1280-compound library of pharmacologically active compounds (LOPAC) was applied as 5 nL/well in 40 columns of one 1536-well black-wall/black-bottom plate, with the remaining 8 columns loaded with 5 nL/well of DMSO, as no-drug controls. For each run of the screen, three plates were loaded with either (1) unlabeled HSR, or (2) HSR pre-incubated with donor-FKBP (D-FKBP; donor-only sample), or (3) donor-only sample that was additionally incubated with 0.3 μM acceptor-CaM (A-CaM) for 60 min prior to loading on the plate (donor-acceptor sample). This sub-saturating concentration of A-CaM was used to provide a readout that is sensitive to library compounds that may increase or decrease CaM binding affinity to RyR. To promote a homogenous population of RyR1 resembling that associated with myopathies, final assay conditions also included 30 nM Ca^2+^ to represent resting (muscle relaxing) Ca^2+^, and 5 mM oxidized glutathione (GSSG), which exaggerates the conditions associated with oxidative stress^32^.

**Figure 1:**
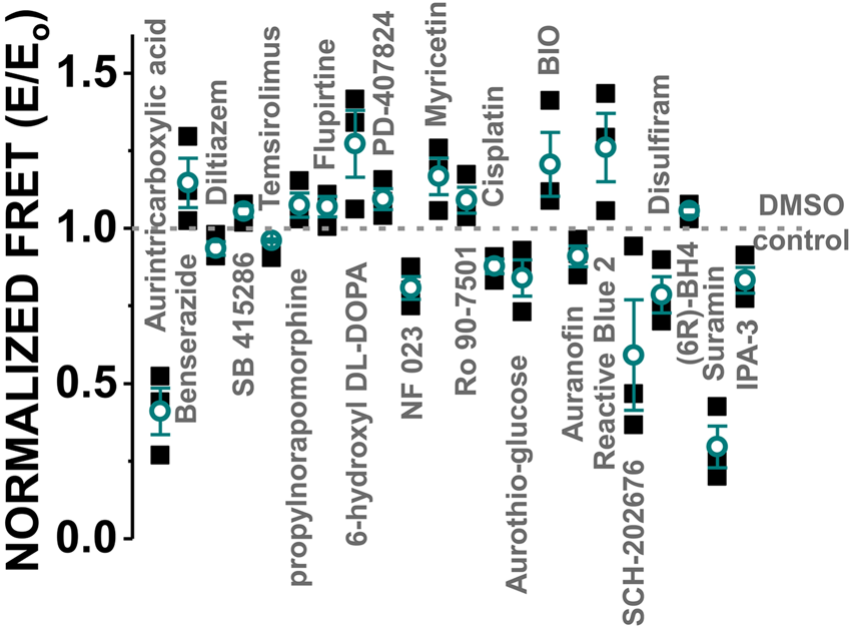
Reproducibility of the FLT-FRET HTS readout. RyR1-specific FRET (E) was measured in the presence of LOPAC compounds (10 μM test-compounds) in 1536-well format. Normalization was relative to DMSO-only controls (E_0_). Hits were those compounds that altered FRET by > 4 SD in at least two of three repeats of the screen. Each data point is shown as a solid black square, and means ± SE are shown in green, n = 3.

**Figure 2:**
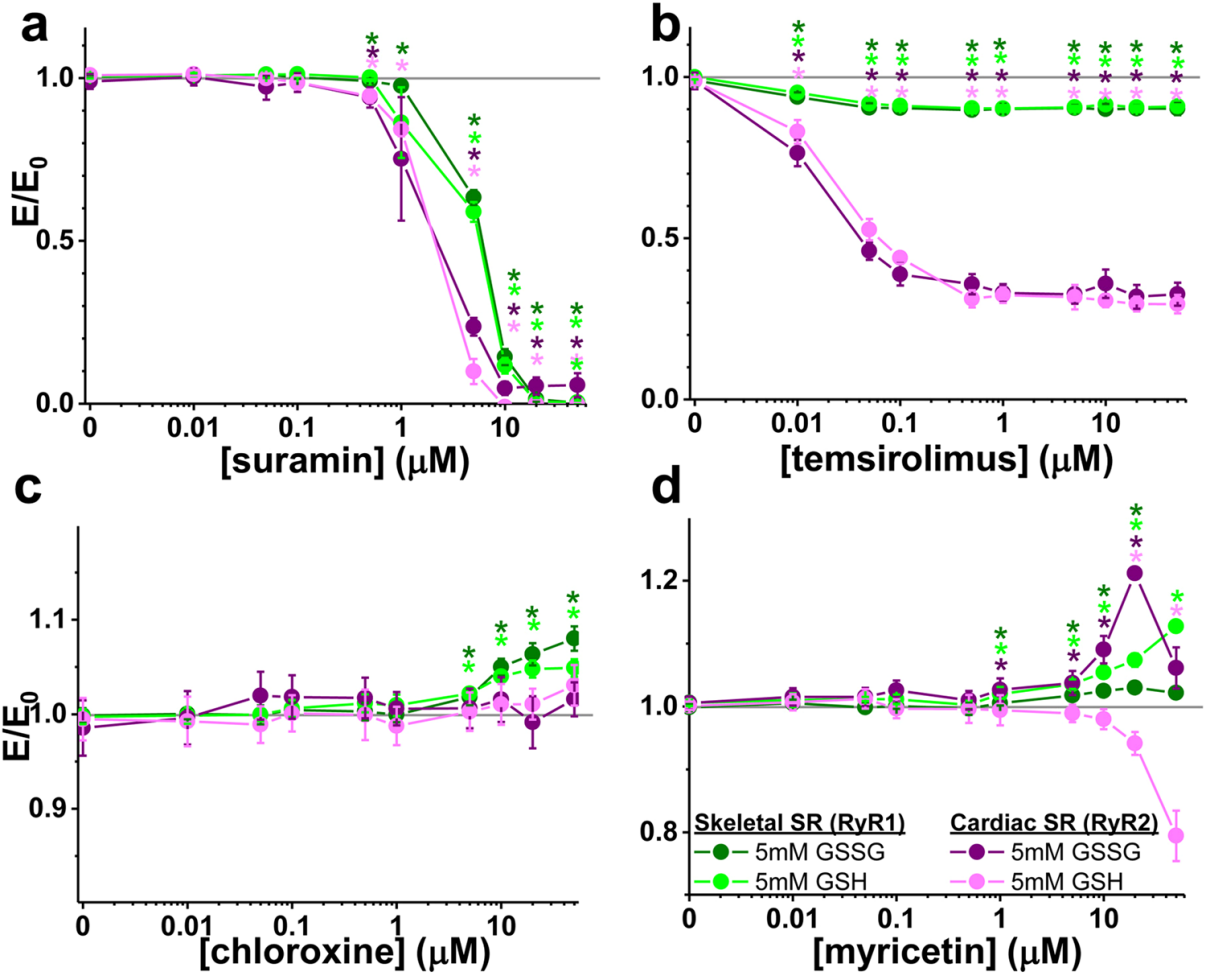
FRET profiles for RyR1 and RyR2 over a range of Hit concentrations. The same FLT-detected FRET readout used in the primary HTS was measured at several Hit concentrations, using RyR1 and RyR2 samples. FRET dose-response of (**a**) suramin, (**b**) temsirolimus, (**c**) chloroxine, and (**d**) myricetin, measured at 30 nM Ca^2+^ using skeletal (green tones) and cardiac (red tones) SR membranes, in the presence of 5 mM GSSG (dark tones) or GSH (light tones). FRET efficiency in the presence of compound (E) was normalized to FRET efficiency in the presence of DMSO-only (E_0_). Data are presented as means ± SEM, n = 4. *P < 0.05 vs. DMSO by 2-sided Student’s unpaired T-test.

We acquired both FLT waveforms and fluorescence spectra, as previously described^19,33,34^. As previously observed^19^, Hit effects were greatest following a 2-hour incubation with the library compounds (Supplementary Fig. 1a). As a result, all FRET results shown in Figs. 1 and 2, and Supplementary Fig. 2 are reported from the 2-hour incubation.

False Hits were filtered out when they altered the donor-only FLT by > 3 SD of the DMSO control mean, and also altered the integrated intensity of the unlabeled HSR spectrum by > 3 SD of the DMSO control.

From the collective of E/E_0_ values, we fit a narrow Gaussian distribution (σ = 0.016) centered over the control mean (μ = 1.004), which indicates that there is negligible bias to increase or decrease FRET (Supplementary Fig. 1b). The Zʹ value uses statistical effect size and signal variation as a gauge of HTS assay quality, with 0.5 ≤ Z′ < 1 indicating an excellent assay that is ready for large-scale HTS^35^. Using 1536-well plates loaded with 10 μM suramin or DMSO (control), we found the Z′ values at the 20 min and 120 min reads were 0.83 ± 0.06 and 0.88 ± 0.12, respectively. This excellent assay quality is very similar to our previous report using 384-well plates (Z′ = 0.89)^19^.

An initial Hit rate that is typically considered to be acceptable for an HTS assay ranges ~0.5–3%^36^. With application of this guideline to our assay, we chose a threshold of four standard deviations (4 SD) of the control_DMSO_ mean, which led to 1.7–2.5% Hit rates in the three runs of the screen. Of the three screens, 22 compounds were Hits in at least two runs (Fig. 1), and 17 compounds were Hits in all three runs (Supplementary Table 1).

The LOPAC chemical collection contains previously established RyR modulators, and we were pleased that our screen identified some of these, including suramin, NF023, and disulfiram (Fig. 1)^19,37,38^. Furthermore, we also identified myricetin and temsirolimus, which are analogues of known RyR modulators quercetin and rapamycin, respectively^39,40^. Indeed, suramin and rapamycin have been used as tools to induce dissociation of fluorescently labeled CaM and FKBP12.0/12.6, respectively^41,42^. Additionally, cisplatin mediates CaM crosslinking at methionine residues^43^, which could explain the decrease in FRET. We were unsurprised to observe “frequent hitters” in screens and thiol modifiers, such as Reactive Blue^44^, SCH-202676^45^, cisplatin^46^, aurintricarboxylic acid^47–49^, and (6 R)-BH4^50^. Several Hits that induce prominent FRET effects and/or are known functional modulators were further tested in dose-response FRET assays. Known RyR or CaM modulators for further testing included suramin, disulfiram, and cisplatin. We did not pursue further RyR assays with NF023, as this compound was previously established to be a less effective RyR modulator than its analogue, suramin^37^. Given that myricetin and temsirolimus are close analogues of known RyR modulators, we proceeded to further investigate whether these Hit compounds would regulate RyR activity or modulation via mechanisms dependent on FKBP or CaM in a similar manner as their analogue RyR modulators.

### FRET dose-response assay

Necessary counter-screens to our pathological RyR1 conditions include evaluating compound effects in healthy conditions and on the cardiac isoform, RyR2. Using the same FRET assay as in HTS, we tested the dose response of key Hits from this screen and the previously HTS-identified RyR1 inhibitor, chloroxine^19^, on healthy (5 mM GSH) and pathological (5 mM GSSG) conditions for both RyR1 in skeletal SR membranes, and RyR2 in cardiac SR membranes.

As previously observed, suramin abolishes FRET by 20 μM (Fig. 2a)^41^. Here, we note that suramin potency (IC50) was not altered by GSH or GSSG. However, we observe an isoform difference, with suramin’s RyR2 potency slightly higher (IC50 was 2.5 ± 0.3 μM with GSH and 2.4 ± 0.6 μM with GSSG) compared to RyR1 (IC50 was 5.9 ± 0.7 μM with GSH and 7.1 ± 0.5 μM with GSSG) (Fig. 2a). Screen Hit temsirolimus reduces FRET in a similar manner as other FKBP12.0/6 ligands, such as tacrolimus (Supplementary Fig. 2a) and rapamycin (a.k.a sirolimus)^41^. Notably, this FRET reduction by temsirolimus is subtle (~10%) for RyR1, but quite robust (68–70%) for RyR2 (Fig. 2b).

Chloroxine is the only RyR1 inhibitor we identified in a previous FRET-based screen of the 727-compound NIH Clinical Collection^19^. Consistent with this study, FRET was increased with micromolar chloroxine (Fig. 2c) for the RyR1 samples. However, chloroxine did not alter FRET with RyR2, indicating an RyR1-specific structural effect for this compound, and suggesting that chloroxine does not interact with RyR2 in a productive manner (Fig. 2c). Isoform specificity is highly desirable for potential therapeutics, and this result demonstrates that our screening assay can identify RyR isoform specific compound modulators.

We found a surprising result with myricetin, one of the LOPAC screen Hits that increases FRET (Fig. 1). Given that quercetin (a myricetin analogue) has been previously shown to increase RyR1 activity^39^, we were expecting that myricetin would also be an activator, and therefore decrease FRET. We found the opposite – myricetin increased the FRET efficiency readout in the primary screens, and the FRET dose-response assays also show that myricetin increases FRET for RyR1 in the presence of GSSG or GSH (Fig. 2d). In contrast, FRET with RyR2 is strongly increased in the presence of GSSG, but strongly decreased in the presence of GSH (Fig. 2d), indicating again an intriguing isoform specificity.

FRET dose-response assays of select LOPAC Hits replicated the effect found in the primary screen. Similar to suramin, aurintricarboxylic acid abolished FRET at compound concentrations ≥ 10 μM (Supplementary Fig. 2b). Several compounds more strongly decreased FRET in the presence of GSSG vs. GSH. These compounds include, cisplatin (Supplementary Fig. 2c), disulfiram (Supplementary Fig. 2d), IPA-3 (Supplementary Fig. 2e) and SCH-202676 (Supplementary Fig. 2g). In contrast, Ro 90–7501 has very similar FRET dose-response effects with GSSG or GSH. However, the Ro 90–7501 dose-responses are biphasic, whereby FRET is increased by sub-micromolar [compound], and decreased by micromolar [compound] (Supplementary Fig. 2f).

The Hit-induced effect on FRET between D-FKBP and A-CaM most likely results from one or the combination of factors, including a shift in CaM and/or FKBP12.6 binding, and/or structure of the RyR complex that shifts the D-A distance. Under the same assay conditions as in the primary screen, we assessed the effect of 10 μM Hit on fluorescent FKBP12.6 (F-FKBP) binding to RyRs, using co-sedimentation with pig skeletal and cardiac SR membranes. Overall, myricetin and suramin did not alter F-FKBP binding to RyR1 or RyR2, while temsirolimus and tacrolimus decreased F-FKBP binding to RyR1 and RyR2 by 20 and 70%, respectively (Supplementary Fig. 3). This suggests that myricetin- and suramin-induced FRET changes are due to shifts in CaM binding rather than shifts in FKBP binding, whereas temsirolimus induced FRET changes are due to shifts in FKBP (rather than CaM) binding, similar to tacrolimus.

### Effect of hits on RyR activity using [^3^H]ryanodine assays

To evaluate the functional impact of compounds identified through our FRET-based screen, we first used [^3^H]ryanodine binding assays. The level of [^3^H]ryanodine binding to RyRs in SR membranes is a well-established index of the RyR channel activity^51^. Given the role of CaM binding in our FRET readout, we investigated whether CaM influences the functional effect of the Hit compounds by carrying out [^3^H]ryanodine binding assays in the absence and presence of CaM (300 nM).

**Figure 3:**
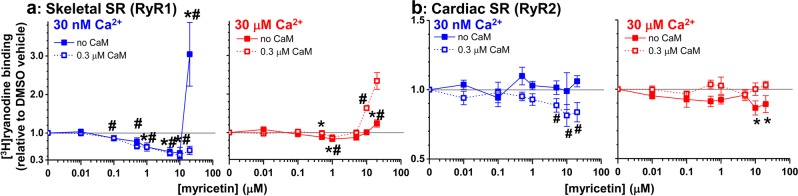
[^3 ^H]ryanodine binding profiles for RyR1 and RyR2 in the presence of a range of myricetin concentrations. Dose-dependent (0–20 μM) effect of LOPAC HTS Hit, myricetin, on [^3^ H]ryanodine binding to skeletal SR (**a**) and cardiac SR (**b**) in the absence of CaM (closed symbol) or in the presence of 300 nM CaM (open symbol), at 30 nM (blue) or 30 μM (red) free Ca^2+^. Data are shown normalized relative to the values for no-drug DMSO control (gray line), means ± SEM, n = 4–6. *P < 0.05 for no-CaM samples vs. DMSO control using 2-sided Student’s unpaired t-test; #P < 0.05 for 0.3 μM CaM samples vs. DMSO control using 2-sided Student’s unpaired t-test.

In our previous study, we identified an inverse correlation between compound effects on FRET and RyR1 activity, as measured by [^3^H]ryanodine binding. In particular, we identified chloroxine, a compound that increases FRET and decreases [^3^H]ryanodine binding to skeletal HSR by 20%^19^. When testing myricetin, shown to increase FRET within the skeletal SR samples here (Fig. 2d, RyR1), we observed a > 50% decrease in [^3^H]ryanodine binding by ≤ 10 μM myricetin, at 30 nM Ca^2+^, with or without CaM (Fig. 3a). However, 20 μM myricetin dramatically increased [^3^H]ryanodine binding, but only in the absence of CaM (Fig. 3a). This biphasic effect is more prominent at 30 μM Ca^2+^, with a maximum ~20% decrease in [^3^H]ryanodine binding reached at 1 μM myricetin, followed by an increase in [^3^H]ryanodine binding at higher myricetin concentrations. The biphasic effect elicited by myricetin in 30 μM Ca^2+^ is observed in both the presence and absence of 0.3 μM CaM (Fig. 3a), although the activation is more robust in the absence of CaM. In contrast to its RyR1 modulatory effects, micromolar myricetin only inhibits [^3^H]ryanodine binding to cardiac SR (RyR2), but this effect reaches significance only in the presence of CaM at 30 nM Ca^2+^ (Fig. 3b, left panel), and only in the absence of CaM at 30 μM Ca^2+^ (Fig. 3b, right panel).

To determine the functional effect of other FRET Hits, we also carried out [^3^H]ryanodine binding assays with ATA and Ro 90–7501. Similar to myricetin, the FRET enhancer Ro 90–7501 inhibited [^3^H]ryanodine binding (Supplementary Fig. 5). Conversely, the FRET inhibitor ATA is a potent inhibitor of [^3^H]ryanodine binding to skeletal SR (Supplementary Fig. 5). This is the first observed exception to the FRET-function correlation.

### Assessing RyR modulators with human mechanically skinned fibers

By using SR membranes in [^3^H]ryanodine binding assays and FRET assays, we have explored the effects of myricetin and chloroxine on a relatively more purified, semi-physiological state of RyR1, in which the EC-coupling apparatus has been largely dismantled. To explore the functional effects of these RyR1 stabilizer compounds on SR Ca^2+^ leak in a physiological system that has an intact EC-coupling apparatus, we used the recently established system of measuring SR Ca^2+^ leak via a confocal microscopy readout of enclosed transverse-tubule [Ca^2+^] in human and rat mechanically skinned skeletal muscle fibers^31^.

**Figure 4:**
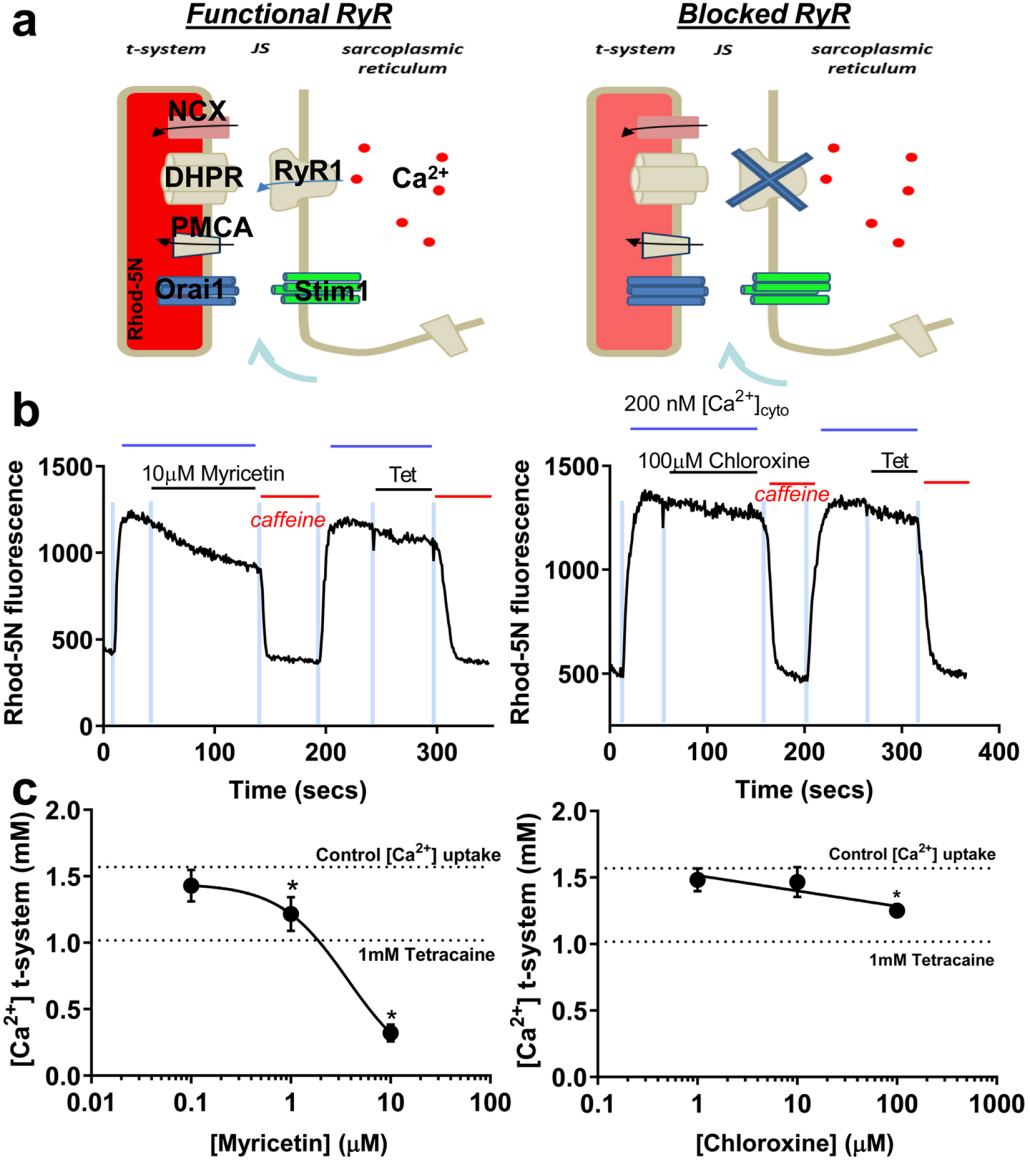
Effects of HTS Hits on t-tubule Ca^2+^ measurements of RyR1 leak in human skinned skeletal muscle fibers. (**a**) Diagram showing how rhod-5N trapped in the sealed t-system can be used to detect RyR Ca^2+^ leak. At rest, Ca^2+^ leaks through the RyR into the junctional space, where it is taken up by the NCX and PMCA, thus resulting in a net increase of [Ca^2+^]t-sys (left). Blockage of the RyR inhibits Ca^2+^ leak into the JS (right). The difference in [Ca^2+^]_t-sys_ under these conditions represents total Ca^2+^ leak^31^. (**b**) Representative traces of t-tubule Ca^2+^ in response to RyR modulators. Solution exchanges are indicated by the blue, vertical bars. The blue horizontal line indicates the presence of 200 nM [Ca^2+^]_cyto_ (standard solution) in the fiber bathing solution, which loads the SR and t-system with Ca^2+^ in the presence of functional RyRs. The functional RyR leaks Ca^2+^ into the junctional space to increase local [Ca^2+^] at the t-system and increase t-system Ca^2+^-dependent fluorescence. The addition of RyR modulators to the standard solution are indicated by the horizontal lines. Each RyR modulator causes a reduction in rhod-5N fluorescence, indicating an effect on RyR Ca^2+^-leak. The red horizontal line indicates the change to a bathing solution with 30 mM caffeine, which causes SR depletion and activation of SOCE to deplete the t-system of Ca^2+^. (**c**) dosage response curve was developed using 0.1, 1 and 10 µM of myricetin (means ± SEM, *P < 0.05, n = 4) and 1, 10 and 100 µM of chloroxine (means ± SEM, *P < 0.05, n = 4–8). The “1 mM Tetracaine” dotted line indicates full RyR inhibition (mean, n = 11). The “Control [Ca^2+^] uptake” dotted line represents no-drug control (full t-system uptake) (mean, n = 15).

As schematically illustrated in Fig. 4a, at 200 nM cytoplasmic Ca^2+^ concentration ([Ca^2+^]_cyto_), RyR1 leaks Ca^2+^ into the junctional space (JS). The magnitude of the leak can be distinguished from the secondary source of Ca^2+^ to the JS, the Ca^2+^ diffusing in from the bulk cytoplasm, by comparing the steady-state [Ca^2+^] in the transverse-tubule system ([Ca^2+^]_t-sys_) in the presence and absence of RyR Ca^2+^ leak, with the use of a RyR blocker tetracaine^31^. The Ca^2+^ entering the JS sets the t-system steady-state level via the activity of PMCAs facing the JS, which is sensitive to nanomolar [Ca^2+^]^31^. The experimental model maintained [Ca^2+^]_cyto_ at 200 nM. This is above resting physiological levels, thus overloading the SR and inducing an increase of RyR Ca^2+^ leak^31^. These conditions allowed the model to resemble that of a pathological nature. In a typical experiment, we use rhod-5N fluorescence to monitor [Ca^2+^]_t-sys_ before and after bath exchange with one concentration of myricetin or chloroxine (Fig. 4b). To determine the total [Ca^2+^]_t-sys_ and the contribution of RyR1 leak to [Ca^2+^]_t-sys_, we exchange the bath solution with 30 mM caffeine and 1 mM tetracaine, respectively (Fig. 4b). Caffeine causes SR depletion and activation of store-operated Ca^2+^ entry (SOCE), which depletes [Ca^2+^]_t-sys_. As shown in Fig. 4b, recovery of the t-system fluorescence after washout of caffeine and re-introduction of standard solution after 10 μM myricetin or 100 μM chloroxine, indicates that the modulators can be washed-out of the fiber.

As shown in Fig. 4c, the presence of 1 μM myricetin (P = 0.0027) and 10 μM myricetin (P < 0.0001) in bath solutions decreased the steady-state [Ca^2+^]_t-sys_ compared to control (upper dotted line), indicating a decrease in RyR1 leak. However, given that the effect of 10 μM myricetin went beyond the effects of 1 mM tetracaine (lower dotted line), a known RyR channel blocker, we can infer that modulation of non-RyR targets leads to the additional decrease in t-system Ca^2+^. This suggests that high concentrations of myricetin inhibit RyR leak but also decrease the activity of the PMCA or open a Ca^2+^ efflux pathway from the t-system to lower [Ca^2+^]_t-sys_.

At all three concentrations tested, chloroxine progressively reduced the steady state towards that of 1 mM tetracaine (Fig. 4, lower dotted line). Only the presence of 100 μM chloroxine in internal solutions statistically decreased the steady-state [Ca^2+^]_t-sys_ (P = 0.0002) (Fig. 4c).

The compound effects on mechanically skinned rat muscle fibers (Supplementary Fig. 6) were very comparable with those observed on human skeletal muscle fibers (Fig. 4). Using the rat muscle fibers, we found that our compound solvent, DMSO, did not alter t-system fluorescence, indicating that 0.1% DMSO does not alter RyR1 Ca^2+^ leak in our model system (Supplementary Fig. 6a).

### Assessing RyR modulators with mechanically skinned fibers

**Figure 5:**
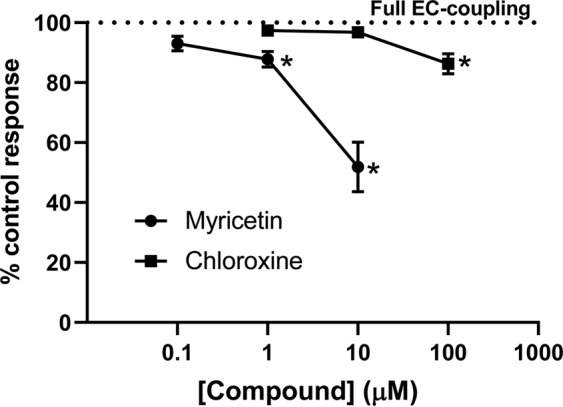
Effect of micromolar myricetin and chloroxine on electrically evoked Ca^2+^ transients. In rat skinned fibers, Ca^2+^ transients were obtained by confocal line scans parallel to the fiber long axis, with corresponding line averaged and normalized rhod-2 fluorescence signals (F/F_0_). Representative recordings of Ca^2+^ transients are shown in Supplementary Fig. 7. All solutions contained 1 mM EGTA and 100 nM free Ca^2+^. Cytosolic Ca^2+^ transients were elicited by electrical field stimulation at 1 Hz in the presence of DMSO (control), myricetin or chloroxine. Dotted line represents 0.1% DMSO control (which causes no detectable reduction in EC-coupling). Data presented as means ± SEM, *P < 0.05, n = 6.

To test the potential impact of these compounds on excitation contraction coupling, we measured voltage-induced Ca^2+^ transients in rat skeletal muscle fibers. To do this, we again used mechanically skinned fibers. Because the t-system reseals upon mechanical skinning, bathing the preparation in a K^+^-based cytoplasmic solution (with a small amount of Na^+^) allows the Na^+^-K^+^ pump to re-establish the normal membrane potential. EC coupling can be initiated by exciting the preparation with field pulses that generate action potentials in the t-system to trigger Ca^2+^ release^52–54^. Ca^2+^ release is tracked by imaging rhod-2 in the cytoplasm via confocal microscopy in line-scan mode, and quantitatively plotted in Fig. 5 (based on recordings such as shown in Supplementary Fig. 7). A major advantage of using skinned fibers is that known concentrations of RyR modulators can be added to the cytoplasm, as they were added in the “leak” experiments. With this approach, we found 1 and 10 μM myricetin reduced Ca^2+^ transient peak amplitude by 12% (P = 0.0046) and 48% (P < 0.0001), respectively (Fig. 5 and Supplementary Fig. 7a). In the case of chloroxine, only addition of 100 μM led to a detectable 14% (P < 0.0001) reduction in the Ca^2+^ transient peak amplitude (Fig. 5 and Supplementary Fig. 7b). Therefore, both compounds, have impact on Ca^2+^ transients that is markedly lower than their effect on the t-tubule readout of RyR1 [Ca^2+^] leak (Figs. 4 and 5).

## Discussion

We have used an FLT-FRET structural readout, of FKBP and CaM binding to RyR1, to identify small-molecule modulators of RyR1 channel function, with a particular interest in identifying compounds that mitigate pathological RyR1 leak in low-nanomolar Ca^2+^ conditions that are typical of the resting muscle sarcoplasm. Through functional validation using [^3^H]ryanodine binding (an *in vitro* biochemical assay), we identified myricetin (this study) and chloroxine (preceding study)^19^ as RyR1 inhibitors. To validate the effectiveness of our RyR-specific FLT-FRET assay (a structural/binding readout) in identification of compounds that inhibit RyR1 channels in their natural context, we demonstrated the mitigating effects of these two compounds on RyR1 Ca^2+^ leak in mechanically skinned human and rat skeletal muscle fibers.

### Assay metrics vs. previous screens

Relative to our previous screening format, we scaled up from 384-well plates to 1536-well plates, which are typically used for large-scale primary screening in the HTS facilities of pharmaceutical companies. This transition reduced the sample consumption by 10-fold (from 50 to 5 μL per well), and increased the data acquisition rate by 40% (from 6583 wells/hour to 9216 wells/hour) without degrading signal window or precision. Thus, the excellent quality of this HTS assay is preserved, as indicated by the industry standard of HTS assay quality, a factor Z′ that is > 0.5. Currently, our throughput (1536- vs. 96-well plates) and HTS assay quality (as indicated by Z′ > 0.8 vs. < 0.5) exceeds all other published screening methods for identifying RyR modulators^30^. In particular, the approach reported by Murayama and colleagues^30^, of monitoring compound effects on ER Ca^2+^ in HEK293 cells, directly detects therapeutically desirable increases in ER Ca^2+^. However, their method does not discriminate between allosteric modulators and channel blockers, whereby channel blockers would be therapeutically undesirable. Neither assay platform (ours or Murayama’s) preserves the excitation-contraction coupling apparatus, which is a clear limitation of these platforms. This is why we utilized structurally intact adult skeletal muscle fibers to validate the Hit effects on RyR1 channel leak in its native context, with intact DHPR interaction and all other membrane-bound components of the EC-coupling apparatus unperturbed.

### FKBP and CaM binding as a readout of Hit interaction with the RyR channel complex

Our previous study indicated an inverse correlation between compound effects on FRET and [^3^H]ryanodine binding (more FRET correlated with less [^3^H]ryanodine binding and vice-versa)^19^. However, the limited number of reproducible Hits (5 compounds) in that screen was insufficient for understanding the mechanism that ties our FRET readout with the impact on RyR function. The number of promiscuous compounds in the LOPAC library was a favorable factor for discovering more compounds that interact with the RyR1 complex (Hits). As hoped, our LOPAC screen identified a greater number of reproducible Hits (Fig. 1), especially compounds that increase FRET (Fig. 1). Several Hits were known RyR modulators or their analogues, and modulated FRET in a fashion that largely reflects our previously identified inverse correlation between FRET and functional effect of compound^19^. Such compounds include suramin, NF023, temsirolimus and myricetin, with the last three compounds being analogues of suramin, rapamycin and quercetin, respectively^37,38,41,42^. As anticipated for a rapamycin analogue, temsirolimus, we found FRET reduced in a manner that correlates with reduced F-FKBP binding. Furthermore, temsirolimus more strongly reduced FRET and F-FKBP binding with RyR2 than to RyR1, which is reminiscent of a recent report that found disruption of FKBP12.0 binding by RyR modulator CLIC2 was stronger with RyR2 than RyR1^55^.

As previously observed, suramin abolished FRET by promoting CaM dissociation, not FKBP dissociation^41^. Similarly, myricetin altered FRET, but did not alter F-FKBP binding (Supplementary Fig. 3), suggesting that the FRET increase is driven by myricetin mediating increased CaM binding to RyR1 and RyR2. Curiously, the functional effect of myricetin on RyR1 was observed in the presence and absence of CaM under nanomolar Ca^2+^, though the effect was subtly affected by the presence of CaM under micromolar Ca^2+^. Myricetin also inhibited RyR2 in nanomolar Ca^2+^ and this effect required CaM. This apparent allosteric interplay of myricetin and CaM binding to RyR is similar to that reported for dantrolene and CaM in binding to RyR2 in cardiomyocytes^22,23,56^.

In the case of several hits, the compound effect was very similar between GSSG and GSH conditions, with the exception of myricetin on RyR2 in cardiac SR membranes, whereby FRET was decreased and increased in GSSG and GSH conditions, respectively (Fig. 2d). This suggests that myricetin may also act on RyR2 by altering posttranslational modifications, which are known to affect the RyR2-CaM interaction^22,23^. In comparison, GSH and GSSG did not elicit significantly different myricetin effects with the RyR1 FRET readout (Fig. 2d). This implies important differences in the myricetin mechanism of action with RyR1 vs. RyR2, in the presence of GSH. Resolving the details of myricetin’s mechanism of action, and its functional impact on RyR2 *in vivo*, will be the focus of separate studies.

As observed in the NIH Clinical Collection screen^19^, it appears to hold true that compounds that increase FRET decrease RyR activity in GSSG conditions. However, we found the inverse does not necessarily hold true, as ATA substantially decreased both FRET and RyR1 activity.

### Testing leak in human skeletal muscle fibers

Our results indicate that the inhibitory effect of chloroxine^19^ and myricetin (Fig. 3) on RyR1 function in skeletal SR membranes can be replicated on RyR1 SR Ca^2+^ leak in human and rat skinned skeletal muscle fibers (Fig. 4, Supplementary Fig. 6). Curiously, the effect of 10 μM myricetin goes beyond the effects of tetracaine, which was used to zero the RyR1 contribution to SR Ca^2+^ leak. This is probably due to myricetin having secondary effects on t-tubule [Ca^2+^]. Notably, reversibility of compound effects suggest that this is not due to shifts in post-translational modifications (Fig. 4b), but rather to a direct effect on t-system Ca^2+^ uptake or release. The versatility of the mechanically skinned fiber preparation^57^ also allowed assessment of RyR1 modulators on action potential induced Ca^2+^ release, as previously demonstrated with dantrolene^54^. Optimally, the ideal RyR1 modulator must mitigate pathological leak but also have minimal effect on normal EC coupling within the muscle. Furthermore, the modulator should have minimal effects on healthy RyR2 isoforms, otherwise healthy cardiac and brain function may be adversely effected. Although not completely ideal, myricetin displays a lot of desirable modulatory effects, including reducing RyR1 activity and SR Ca^2+^ leak, while having minimal functional effects on RyR2 activity. Thus, myricetin may be a test candidate for treatment of skeletal muscle disease animal models, including MD and sarcopenia.

Overall, information provided by these skinned skeletal muscle fiber assays is important because it identified RyR Ca^2+^ leak inhibitors with potential for lead compound development, showed concentration dependent reductions in SR [Ca^2+^] leak, showed non-specific effects of modulators, and most importantly for clinical relevance, showed the effects on EC coupling.

### Strengths and limitations of the primary screening assay

**Figure 6:**
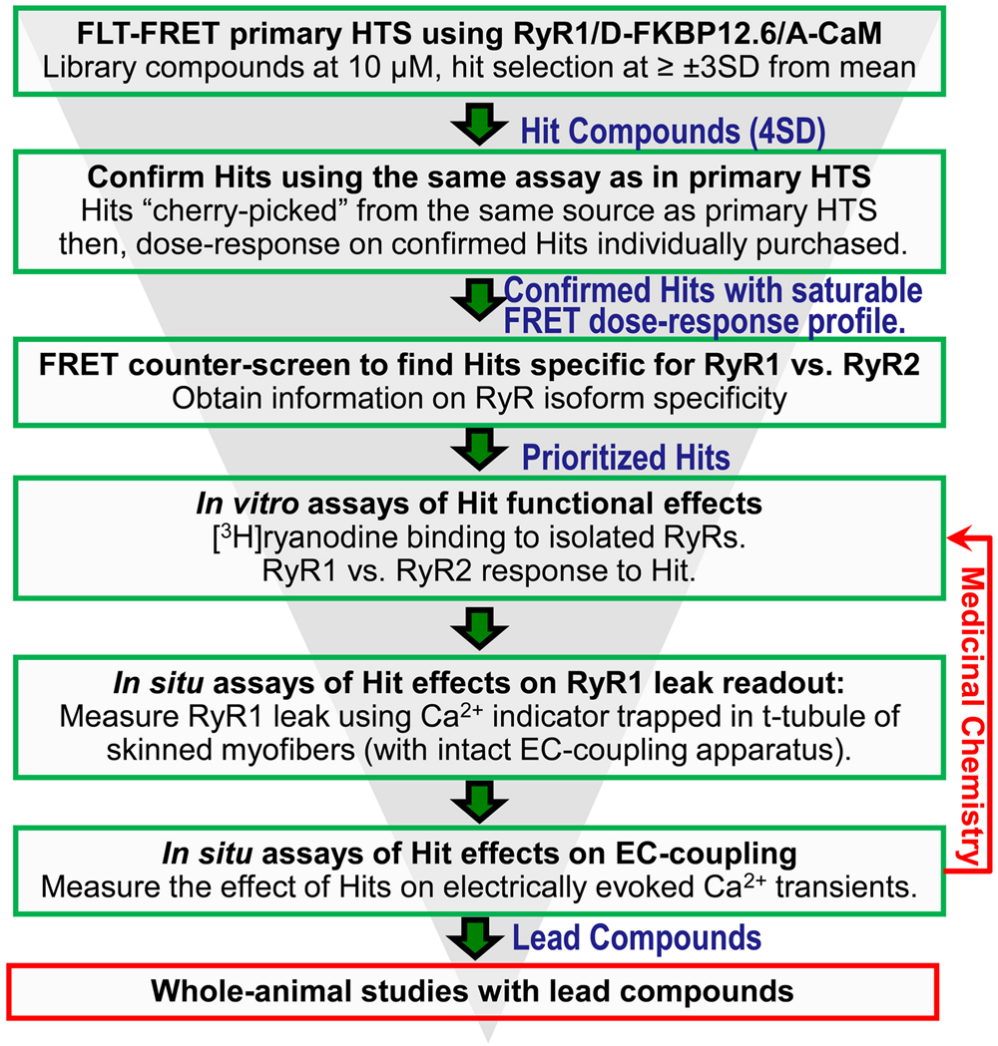
RyR1 Drug Discovery Pipeline. Green boxes denote early-stage steps (assays) in the RyR1 drug discovery pipeline that are covered in this report. In red, we summarize additional steps that will be necessary in a full-scale drug discovery campaign.

We describe a target-focused primary HTS assay that uses a high-precision FLT-FRET readout that is specific to the RyR. Target identification is critically facilitated via binding of donor-labeled FKBP12.6 to its sole partner in sarcoplasmic reticulum – the RyR, and then observing FLT changes due to binding of acceptor-labeled CaM within FRET range of the donor. Thus, by looking under the donor lamp post we obtain a signal exclusively corresponding to A-CaM binding to RyR, as opposed to A-CaM binding to other targets in SR. This is essential to demonstrating RyR engagement at the earliest steps of the screening funnel (Fig. 6**)**. Demonstration of target engagement represents a significant challenge in the case phenotypic HTS platforms, such as described by Murayama and colleagues^30^. A limitation of structure-based, target-focused HTS assays, such as ours, may be that functional validation of the Hits occurs in downstream studies that could be resource-intensive. To address this limitation, we use a structural readout of CaM-RyR binding that has been strongly correlated to functional effects^19,22,23,26^. As discussed above, this correlation holds for most (but not all) Hits, meaning that the direction of FRET change is not a perfect predictor of the functional effect of a Hit. Compounds that bind to the target (RyR) and do not affect its properties in a manner that affects the structural readout will go undetected (false negatives). This is a typical limitation of target-based HTS assays in general, i.e., not all desirable effectors will be picked up. For an enormous target such as the largest known channels, the RyRs, one could hypothesize that some small-molecule modulators could bind without an effect on the FRET readout. This is especially if they bind to RyR structural elements situated between the FKBP or CaM binding site and the channel pore, and do not produce retrograde structural rearrangements that affect FKBP or CaM binding or their distance relationships. We view this as a testable basic-science hypothesis. However, recent structural studies have revealed well-defined structural pathways for allostery connecting the outskirts of the RyR cytosolic cap to modulate the transmembrane pore opening^58^. Moreover, we feel that our readouts – RyR binding of CaM or FKBP – are well documented as linked to the RyR functional state, and their disrupted binding has been consistently observed in pathological states^19–23,26,28,29,59,60^.

In conclusion, we describe a pipeline for early-stage RyR1-targeted drug discovery by demonstrating that the FLT-FRET structural readout of our HTS-compatible platform can identify compounds that inhibit a biochemical index of RyR channel function (the [^3^H]ryanodine binding assay), as well as RyR1 leak in skinned human and rat skeletal muscle fibers. We also use muscle fibers to evaluate the effect of compounds on EC coupling, as evidenced by voltage-induced Ca^2+^ transients. The compounds identified by our FRET-based HTS assay and further tested on skeletal muscle fibers are unlikely to be considered potential therapeutics, but they can represent starting points for lead development with iterations of testing analogue compounds generated by medicinal chemists. Immediate future work involves screening larger (50,000-compound) libraries, followed by lead development. Structure-function correlations enabled by biochemical, cell physiology, and spectroscopic studies in combination with recent advances in high-resolution RyR cryo-EM^25,58,61–66^ are expected to resolve the molecular mechanism of action for RyR modulators emerging from reports such as this one.

## Methods

### Compound handling and preparation of 1536-well assay plates

The LOPAC compounds (Sigma-Aldrich, MO, USA) were received in 96-well plates and reformatted into assay plates as previously detailed^67,68^. Assay plates were prepared by transferring 5 nL of the 10 mM compound stocks in columns 3–22 and 27–46 or DMSO in columns 1–2, 23–26 and 47–48 from the source plates to 1536-well black polypropylene plates using an Echo 550 acoustic dispenser. These assay plates were stored at −20 °C prior to usage.

### Isolation of SR vesicles

Crude sarcoplasmic reticulum (CSR) vesicles were isolated from porcine *longissimus dorsi* muscle and porcine cardiac left ventricle tissue by differential centrifugation of homogenized tissue^69^. HSR vesicles, which are enriched in RyR1, were isolated by fractionation of crude skeletal SR vesicles using a discontinuous sucrose gradient^69^. All vesicles were flash-frozen and stored at −80 °C. Immediately prior to the fluorescence or [^3^H]ryanodine binding studies described below, the SR vesicles were stripped of residual endogenous CaM by incubation with a peptide derived from the CaM binding domain of myosin light chain kinase, followed by sedimentation^70^.

### Expression, purification and labeling of FKBP and CaM

Single-cysteine mutants of FKBP12.6 (C22A/T85C/C76I) and CaM (T34C) were expressed in *Escherichia coli* BL21(DE3)pLysS (Agilent Technologies, CA, USA), purified and were respectively labeled with fluorescent probes AF488 or AF568 as described previously^51,71^. We have previously demonstrated that our system of donor-labeling FKBP12.6 (D-FKBP) with AF488 and acceptor labeling CaM (A-CaM) with AF568 does neither alter RyR binding nor modulatory activity of these proteins^71,72^.

### Preparation of SR vesicles for FRET measurement

Skeletal HSR and cardiac CSR (0.4 mg/ml) membranes were independently pre-incubated with 60 nM D-FKBP, for 90 min, at 37 °C, in a solution containing 150 mM KCl, 5 mM GSH, 0.1 mg/mL BSA, 1 µg/mL Aprotinin/Leupeptin, 1 mM DTT and 20 mM PIPES (pH 7.0). To remove unbound D-FKBP, the SR membranes were spun down at 110,000 × *g* for 20 min, and then resuspended to 1 mg/mL (skeletal HSR) and 2 mg/mL (cardiac CSR) in binding buffer consisting of 150 mM KCl, 5 mM GSSG, 0.1 mg/mL BSA, 1μg/mL Aprotinin/Leupeptin and 20 mM PIPES, pH 7.0. These samples were then incubated with indicated [A-CaM] for 30 min at 22 °C in binding buffer containing 0.065 mM CaCl_2_ to give 30 nM free Ca^2+^ in the presence of 1 mM EGTA (calculated by MaxChelator). This labeled SR sample was applied to the assay plates in 5 μL aliquots using a Multidrop™ Combi reagent dispenser (Thermo Scientific) with a standard-tube. An additional set of the assay plates were loaded with skeletal HSR or cardiac CSR labeled with only D-FKBP (no added A-CaM).

### Fluorescence data acquisition

FLT measurements were conducted in a top-read FLT-PR designed and built by Fluorescence Innovations, Inc^33^. AF488 donor fluorescence was excited with a 473-nm microchip laser from Concepts Research Corporation (Belgium, WI), and emission was acquired with 490-nm long-pass and 517/20-nm band-pass filters (Semrock, Rochester, NY).

### HTS data analysis

FLT waveforms for each well were fit based on a one-exponential decay function using least-squares minimization global-analysis software (Fluorescence Innovations, Inc.), as previously described^19,33^. FRET efficiency (*E*) was determined as the fractional decrease of donor FLT (τ_D_), due to the presence of acceptor fluorophore (τ_DA_), using the following equation:

1 $$E=1-\frac{{\tau }_{DA}}{{\tau }_{D}},$$

HTS assay quality was determined based on FRET assay samples in wells pre-loaded with control (DMSO) and suramin (20 μM final concentration), as indexed by the Z′ factor:^35^

2 $$Z^{\prime} =1-3\left(\frac{{\sigma }_{DMSO}+{\sigma }_{suramin}}{|{\mu }_{suramin}-{\mu }_{DMSO}|}\right)$$

where σ_DMSO_ and σ_suramin_ are the SDs of the control τ_DA_ and suramin τ_DA_, respectively; μ_DMSO_ and μ_suramin_ are the means of the control τ_DA_ and suramin τ_DA_, respectively. A compound was considered a Hit if it changed *E* by > 4 SD relative to that of control samples (E_0_) that were exposed to 0.1% DMSO.

### [^3^H]ryanodine binding to SR vesicles

In 96-well plates, HSR vesicles (1 mg/ml) and cardiac CSR vesicles (3 mg/mL) were pre-incubated with 0.02% DMSO or Hit compound, with or without 300 nM CaM, for 30 min at 22 °C in a solution containing 150 mM KCl, 5 mM GSSG, 1 µg/mL Aprotinin/Leupeptin, 1 mM EGTA, and 65 µM or 1.02 mM CaCl_2_ (as determined by MaxChelator to yield 30 nM or 30 μM of free Ca^2+^, respectively), 0.1 mg/mL BSA and 20 mM K-PIPES (pH 7.0). Non-specific and maximal [^3^H]ryanodine binding to SR were separately assessed by addition of 40 µM non-radioactively labeled ryanodine or 5 mM Adenylyl-imidodiphosphate, respectively. Such control samples were each distributed over 4 wells/plate. Binding of [^3^H]ryanodine (10 and 15 nM for cardiac and skeletal SR, respectively) was determined after a 3-h incubation (37 °C) and filtration through grade GF/B Glass Microfiber filters (Brandel Inc., Gaithersburg, MD, US) using a Brandel Harvester. In 4 mL of Ecolite Scintillation cocktail (MP biomedicals, Solon, OH, USA), [^3^H] on filter was counted using a Beckman LS6000 scintillation counter (Fullerton, CA).

### Muscle preparation for single-fiber imaging

All experiments were approved by The University of Queensland (UQ) Human Ethics Committee, and were performed in accordance with the relevant guidelines and regulations. Subjects signed informed consent forms prior to be their involvement in this study. Human muscle biopsies were collected under local anesthesia from the *vastus lateralis* (VL) muscle, as previously described^73^. All subjects who volunteered in this study were fit and in good physical condition at the time of biopsy. Muscle biopsies were collected under local anesthesia (Xylocaine, 10 mg ml^−1^ with adrenaline, 5 µg ml^−1^) from the mid-portion of VL, using a 6 mm Bergstrom biopsy needle modified for manual suction^74^. Muscle tissue collected from the biopsy needle was blotted on filter paper (Whatman No 1) to remove blood and external fluid. Muscle tissue was pinned to Sylgard set in a petri dish containing paraffin oil.

All experimental methods using rats were approved by The Animal Ethics Committee at The University of Queensland. Five month old Wistar rats (UQ Biological Resources, Brisbane) were euthanized by asphyxiation via CO_2_ exposure, and the *extensor digitorum longus* (EDL) were rapidly excised.

Bundles of fibers were isolated and exposed to a physiological solution containing 145 mM NaCl, 3 mM KCl, 2.5 mM CaCl_2_, 2 mM MgCl_2_, 2.5 mM rhod-5N salt, 0.05 mM BTS (Calbiochem) and 10 mM HEPES (pH 7.4). Bundles were allowed 15 min to equilibrate with the physiological solution and then individual fibers were isolated and mechanically skinned. Skinned fibers with fluorescent dye trapped in the t-system were mounted on a custom-made chamber that used a coverslip as a base and bathed in a standard internal solution, which contained 50 mM EGTA, 90 mM HEPES, 126 mM K^+^, 36 mM Na^+^, 8 mM ATP, 1 mM Mg^2+^, 10 mM creatine phosphate and 200 nM Ca^2+^. For each experimental model, SR and t-system Ca^2+^ was released with 30 mM caffeine in an internal solution with 0.01 mM Mg^2+^. RyR modulators myricetin, chloroxine (HTS ‘Hits’) and tetracaine (known RyR inhibitor) were dissolved as stock solutions in DMSO and added to internal solutions (< 0.1% DMSO).

### Confocal imaging for RyR1 leak in skeletal muscle fibers

Mounted skinned fibers were imaged using an Olympus FV1000 confocal microscope equipped with an Olympus 0.9NA 40x Plan-Apochromat objective. Rhod-5N was excited with 543-nm HeNe laser and the emission was filtered using the Olympus spectra detector. For tracking Ca^2+^ movements across the t-system membrane, images were continuously recorded in xyt mode with an aspect ratio of 256 × 512, with the long aspect of the image parallel with that of the preparation. Temporal resolution of imaging in this mode where the fluorescence signal was within the borders of the fiber was 0.8 s.

T-system rhod-5N fluorescence was converted to [Ca^2+^]_t-sys_ as previously described^75^:

$${[C{a}^{2+}]}_{t-sys}(t)=Kd\frac{(F(t)-{F}_{min})}{({F}_{max}-F(t))}$$

[Ca^2+^]_t-sys_ = concentration of ionized calcium concentration in the t-system, (t) = point in time, K_d_ = dissociation constant of the dye/Ca^2+^ complex, F = fluorescence intensity (arbitrary units) taken at the point of plateau after a solution is applied, F_max_ = maximum fluorescence, F_min_ = minimum fluorescence. The K_d,_(Ca^2+^) of rhod-5N was determined previously^75^ as 0.8 mM.

To determine the effect of RyR modulators on the RyR Ca^2+^ leak, isolated mechanically skinned fibers were continuously imaged as described above, to obtain a record of t-system rhod-5N fluorescence changes over time as the cytoplasmic solution bathing the fiber was changed. SR and t-system Ca^2+^ was released by bathing the fiber in a solution where free [Mg^2+^] was lowered from 1 to 0.01 mM in the presence of 30 mM caffeine (“release solution”). This chronically opened the RyR, thus thoroughly depleting the SR of Ca^2+^ and consequent depletion of t-system Ca^2+^ occurred via the activation of SOCE. Alternatively, the application of 200 nM [Ca^2+^]_cyto_ standard internal solution allowed the uptake of Ca^2+^ and the [Ca^2+^]_t-sys_ reached a steady-state that was partially dependent on Ca^2+^ leaking through RyRs into the tight junctional space between the t-system and SR membranes^31^. By blocking the RyR with tetracaine (1 mM), it was possible to separate the influence of RyR Ca^2+^ leak on t-system Ca^2+^ steady-state from the level determined by the lower concentration of bulk cytoplasmic Ca^2+^ that could otherwise enter the junctional space. This difference provides a measure of RyR Ca^2+^ leak, or a reference for Ca^2+^ leak that we can use to assess the effectiveness of novel RyR modulators in a human muscle fiber. Myricetin (0.1, 1 and 10 μM), chloroxine (1, 10 and 100 μM) or DMSO control (0.1%) were added to the standard internal solution bathing the skinned fibers, at known concentrations, to assess their influence on RyR Ca^2+^ leak.

### Electrically evoked Ca^2+^ transients in rat skeletal muscle fibers

Skinned rat fibers were prepared and bathed in a resting physiological solution containing 100 nM Ca^2+^, 1 mM Mg^2+^, 36 mM Na^+^, 126 mM K^+^, 1 mM EGTA, 90 mM HEPES, 5 mM rhod-2, 10 mM creatine phosphate and 8 mM ATP (pH 7.4). The Ca^2+^ sensitive dye rhod-2 was used to track cytosolic Ca^2+^ transients when exposed to electrical stimulation. Field pulses at 1 Hz frequency and 4 ms in duration were applied across platinum electrodes parallel to the long axis of the fiber as previously described^54,76^. Imaging for electrical stimulation experiments was in xt mode, at a rate of 2 ms·line^−1^. Myricetin (0.1, 1 and 10 μM), chloroxine (1, 10 and 100 μM) or DMSO control (0.1%) were added to the physiological solution. Electrical stimulation recordings were taken no more than 1 min post application of modulator.

### Analysis and presentation of data

We report data as means ± SEM. To determine statistical difference, we used 2-sided Student’s unpaired T-test or one-way ANOVA followed by Tukey’s post-hoc test, as indicated. We used GraphPad Prism and OriginLab Origin software packages to perform these statistical analyses were performed with. Significance was accepted at P < 0.05. IC50 values were derived from dose-response fits to the Hill function.

## Supplementary information

Supplementary Information.

## Data Availability

The authors declare that all data supporting the findings of this study are available within the article and its supplementary information file.
