# Supplementary material for: RyR1-targeted drug discovery pipeline integrating FRET-based high-throughput screening and human myofiber dynamic Ca2+ assays
Source: Sci Rep. 2020 Feb 4;10:1791. doi: 10.1038/s41598-020-58461-1 (PMC7000700; doi:10.1038/s41598-020-58461-1)
Supplement: Supplementary file 1 — Supplementary Information. [file 41598_2020_58461_MOESM1_ESM.pdf]

SUPPLEMENTARY MATERIALS

RyR1-targeted drug discovery pipeline integrating FRET-based high-throughput screening and human myofiber dynamic Ca^{2+} assays

Robyn T. Rebbeck, Daniel P. Singh, Kevyn A. Janicek, Donald M. Bers, David D. Thomas, Bradley S. Launikonis, and Razvan L. Cornea

Supplementary Table 1. Reproducibility and percentage of total Hits for each LOPAC screen. Numbers of Hits yielded by each screen run at 4 SD thresholds.

Screen run number	**1**	**2**	**3**
Hits	27	34	23
Reproducible between 2 screens	21 (78%)	22 (65%)	18 (78%)
Reproducible between 3 screens	17 (63%)	17(50%)	17(74%)

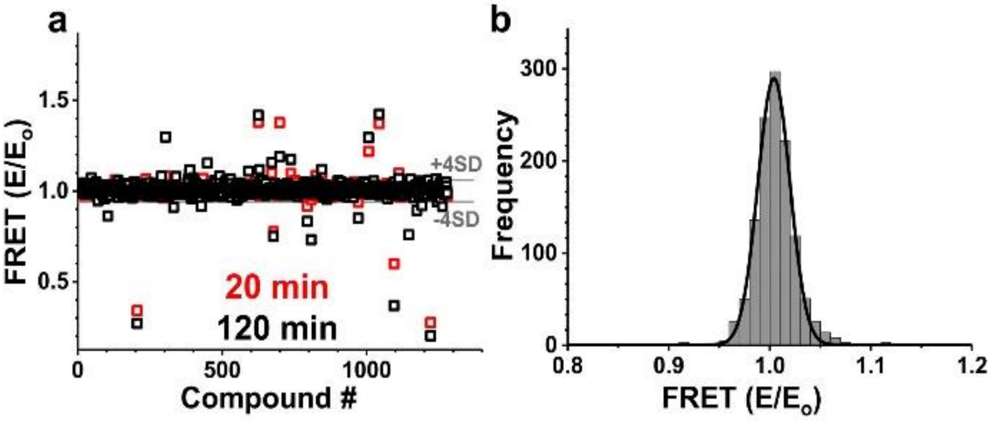

Supplementary Fig. 1 Performance of FLT-detected FRET screen. (a) FLT-detected FRET readouts from RyR-targeted HTS of LOPAC performed in 1536-well format (10 μM final test-compound concentration in each well). All fluorescent compounds that interfere with donor-only FLT were removed from the plotted data set. (b) Gaussian fit of E/E_0 distribution.

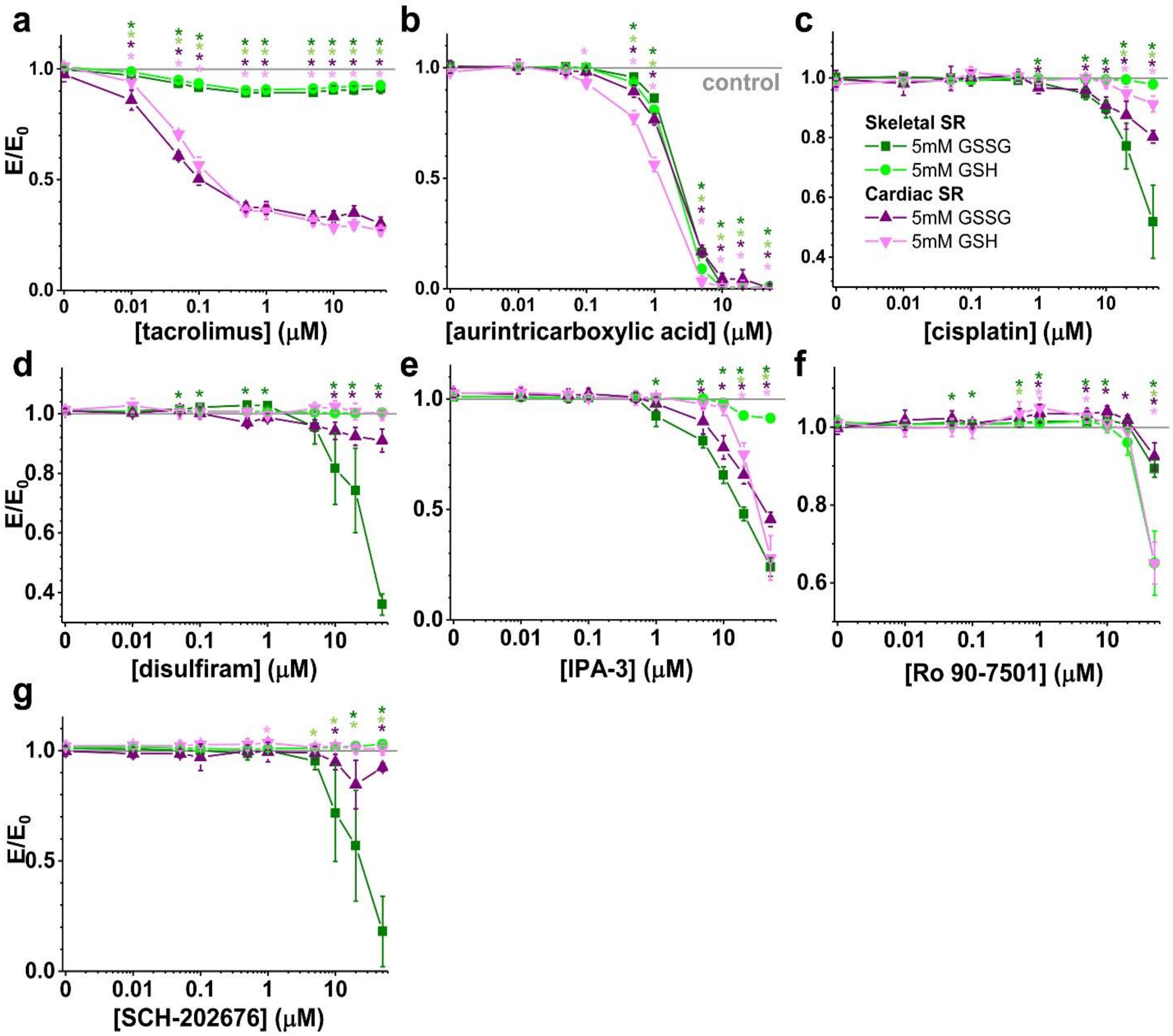

Supplementary Fig. 2 FLT-FRET profiles for RyR1 and RyR2 over a range of Hit concentrations. FLT-FRET dose-response to (a) tacrolimus, (b) aurintricarboxylic acid, (c) cisplatin, (d) disulfiram, (e) IPA-3, (f) Ro 90-7501, and (g) SCH-202676, using pig skeletal HSR (green) and cardiac CSR (purple), at 30 nM Ca^{2+}, in the presence of 5 mM GSSG (dark color) or GSH (light color). FRET efficiency in the presence of compound (E) was normalized to FRET efficiency in the presence of DMSO-only (E_0). Data are presented as means ± SEM, n=4. *P < 0.05 vs. DMSO by 2-sided Student's unpaired T-test.

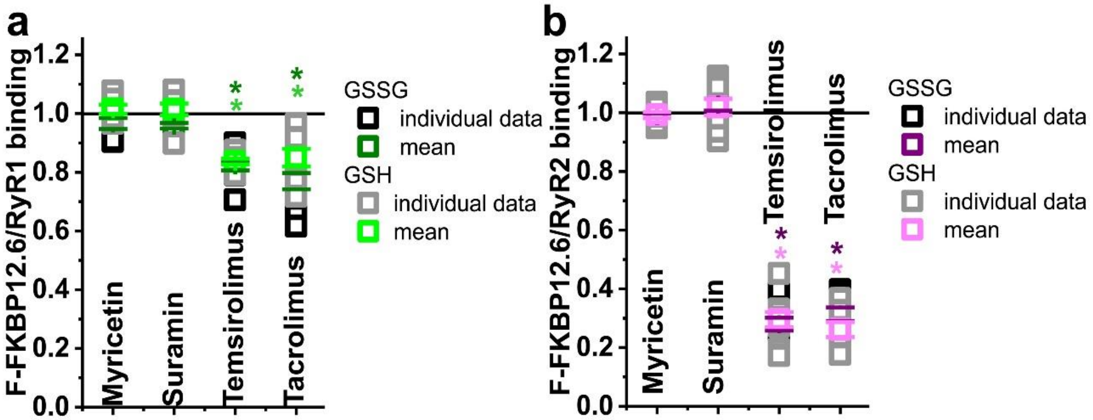

Supplementary Fig. 3 Effect of HTS Hits on F-FKBP binding to a) RyR1 in skeletal HSR membranes, and b) RyR2 in cardiac CSR membranes at 30 nM Ca^{2+} in the presence of 5 mM GSSG or GSH. Results are represented as individual data points and means ± SEM, n = 4-5. *P < 0.05 by 2-sided Student's unpaired t-test.

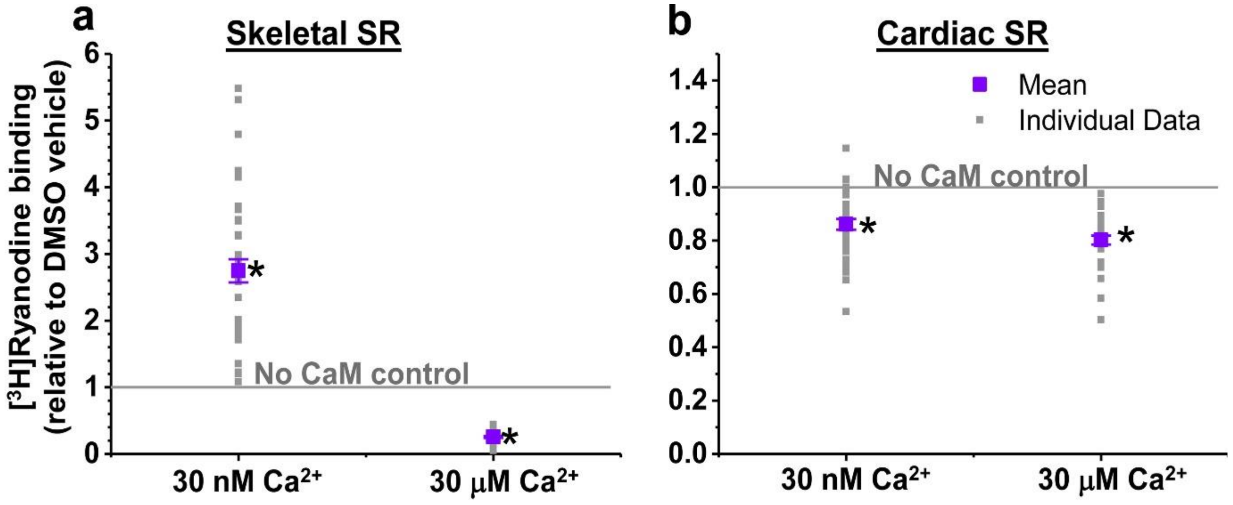

Supplementary Fig. 4 Effect of 300 nM CaM on [^{3}H]ryanodine binding measurements of RyR activity. [^{3}H]ryanodine binding to skeletal SR (a) and cardiac SR (b) in the absence of CaM or in the presence of 300 nM CaM, at 30 nM or 30 μM free Ca^{2+}. Individual data (gray squares) are shown normalized to the values for the no-CaM control (gray lines). Means ± SEM (purple square), n=24-33. *P < 0.05 vs. no-CaM control using a 2-sided Student's unpaired t-test.

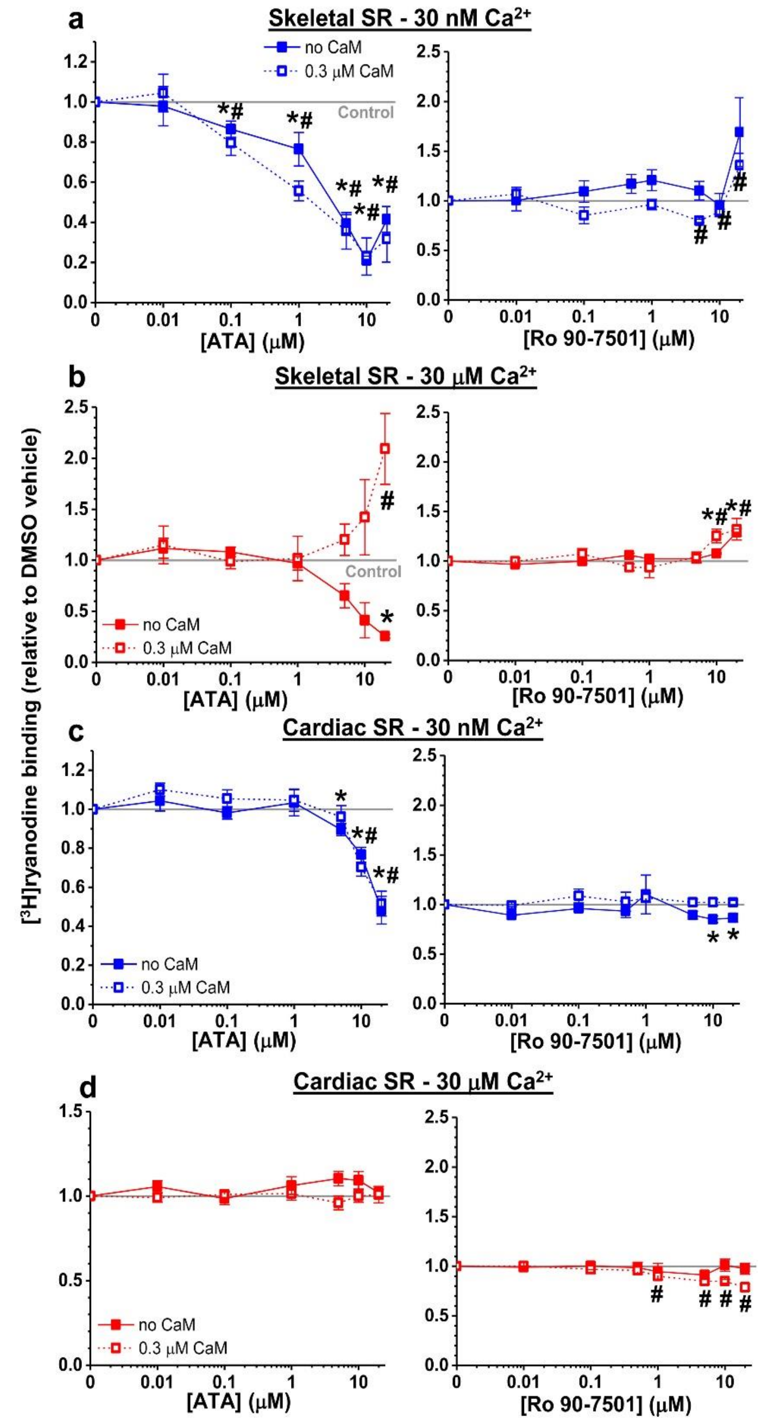

Supplementary Fig. 5 [^{3}H]ryanodine binding dose-response to HTS Hits. Dose-dependent (0-20 μM) effect of LOPAC HTS Hits, Aurintricarboxylic acid (ATA; left) and Ro 90-7501 (right), on [^{3}H]ryanodine binding to skeletal SR (a,b) and cardiac SR (c,d), in the absence of CaM (closed symbol) or in the presence of 300 nM CaM (open symbol), at 30 nM (a,c) or 30 μM (b,d) free Ca^{2+}. Data are shown normalized to the values for no-drug DMSO control (gray line), mean ± SEM, n=4-6. *P < 0.05 for no-CaM sample vs. DMSO control using a 2-sided Student's unpaired t-test. $^{\#}$P < 0.05 for 0.3 μM CaM samples vs. DMSO control using a 2-sided Student's unpaired t-test.

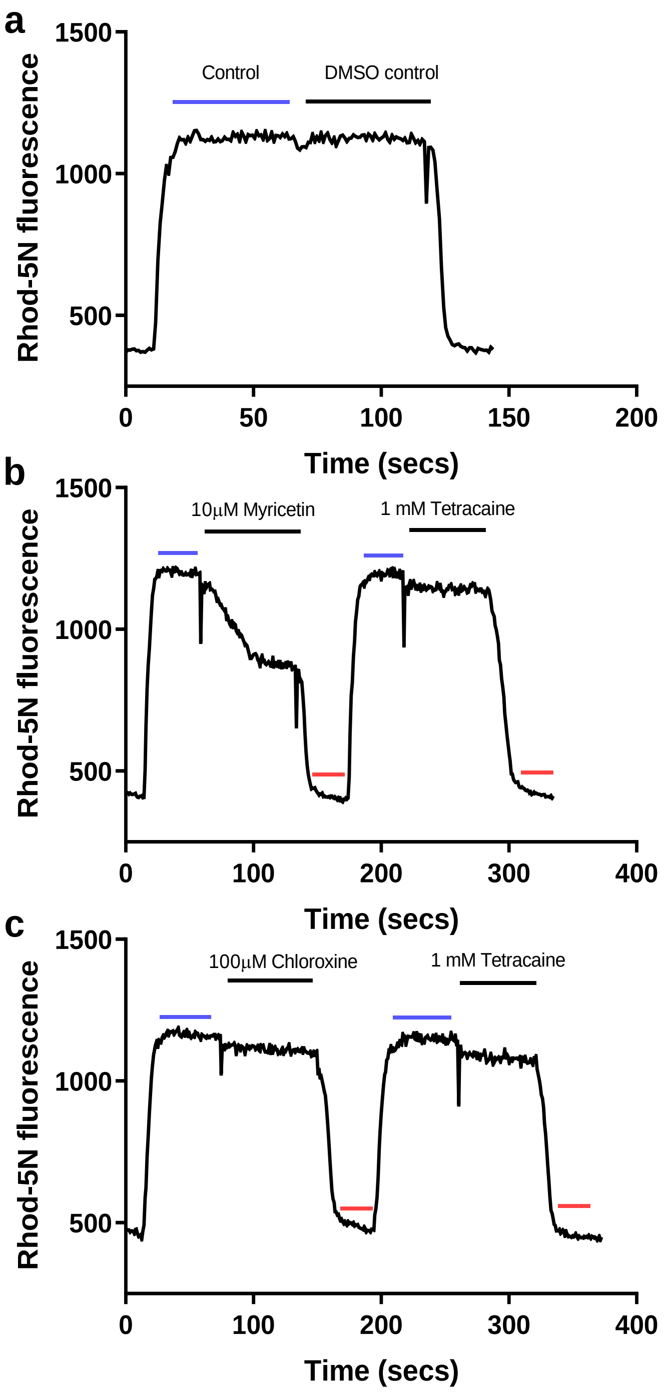

Supplementary Fig. 6 Representative traces for effects of DMSO control and HTS hits on RyR1 leak in rat skinned skeletal muscle fiber. (a) fiber was monitored before and after addition of DMSO control (blue then black bar). (b) fiber exposed to standard solution in the absence, then presence of 10 μM myricetin (blue then black bar); then to caffeine (red bar), to cause thorough depletion of t-sys Ca^{2+}; then to standard solution in the absence and presence of tetracaine (blue then black bar). (c) fiber exposed to standard solution in the absence, then presence of 100 μM chloroxine (blue then black bar); then to caffeine (red bar), to cause thorough depletion of t-sys Ca^{2+}; then to standard solution in the absence and presence of tetracaine (blue then black bar).

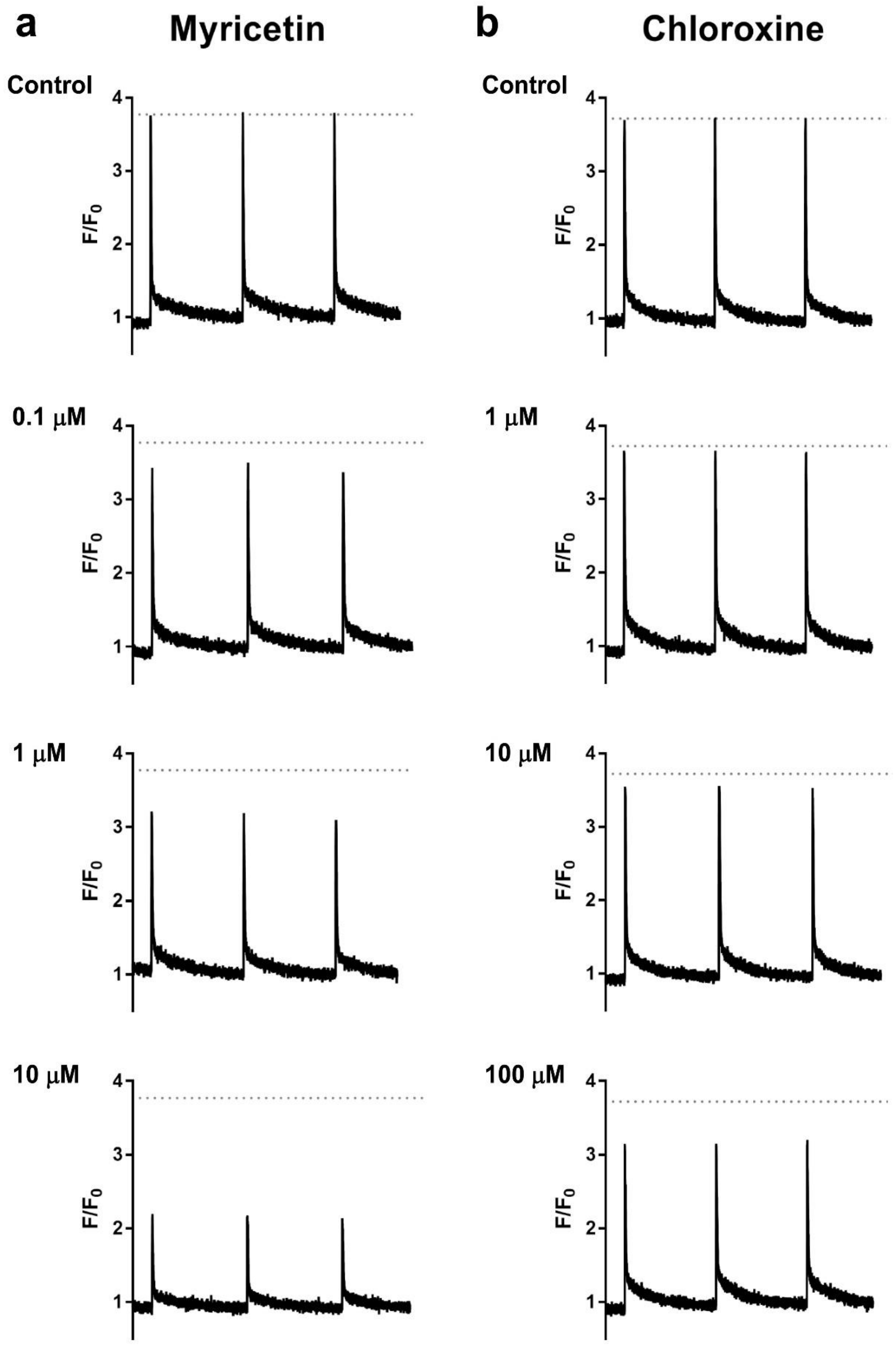

Supplementary Fig. 7 Effect of myricetin and chloroxine on electrically evoked Ca^{2+} transients. Representative Ca^{2+}-transient recordings in rat skinned fibers exposed to 0-10 μM myricetin (a) and 0-100 μM chloroxine (b), as obtained by confocal line-scans parallel to the fiber long axis, with corresponding line averaged and normalized rhod-2 fluorescence signals (F/F_0). Cytosolic Ca^{2+} transients were elicited by electrical field stimulation at 1 Hz in the presence of DMSO (control), myricetin or chloroxine. All solutions contained 1 mM EGTA and 100 nM free Ca^{2+}. The quantitative summary of this data is shown in Fig. 5.
